# TUG-Defined Mobility Impairment and Physical Performance in Adults with Type 2 Diabetes Mellitus

**DOI:** 10.3390/jcm15176787

**Published:** 2026-09-01

**Authors:** Madawi Aljawaee, Neah Albasha, Rakan I. Nazer, Ali M. Albarrati

**Affiliations:** 1 Department of Rehabilitation Sciences, College of Health and Rehabilitation Sciences, Princess Nourah bint Abdulrahman University, Riyadh 11671, Saudi Arabia; maaljawaee@pnu.edu.sa (M.A.); nmalbasha@pnu.edu.sa (N.A.); 2 Department of Cardiac Sciences, College of Medicine, King Saud University, Riyadh 11451, Saudi Arabia; raknazer@ksu.edu.sa; 3 Department of Rehabilitation Sciences, College of Applied Medical Sciences, King Saud University, Riyadh 11433, Saudi Arabia

**Keywords:** type 2 diabetes mellitus, frailty, Timed Up and Go, handgrip strength, six-minute walk test, physical performance

## Abstract

**Background/Objectives:** Type 2 diabetes mellitus (T2DM) is increasingly recognised as a risk factor for physical frailty, driven by sarcopenia, microvascular disease, and chronic hyperglycaemia. Simple, low-cost performance tests may offer a practical means of identifying frailty risk in this population, but data combining several such measures in T2DM remain limited. This study aimed to characterise physical performance and quantify the proportion of adults with T2DM meeting thresholds for low physical performance. Methods: In this cross-sectional study, physical performance was assessed in adults with T2DM using the Timed Up and Go (TUG) test, handgrip strength (HGS), and the six-minute walk distance (6MWD). TUG cutoffs of 8 and 10 s were applied as exploratory external reference thresholds, consistent with cutoffs previously applied in chronic disease and older-adult populations. **Results:** Participants had a mean age of 55.6 ± 4.8 years; 42 (64.6%) were male, and mean BMI was 29.6 ± 4.4 kg/m^2^. Mean TUG time was 9.30 ± 3.02 s, right and left HGS were 30.8 ± 10.1 kg and 28.0 ± 9.3 kg, and mean 6MWD was 401.2 ± 96.3 m. Forty-four participants (67.7%; 95% CI 55.6–77.8) exceeded the 8 s threshold, and 16 (24.6%; 95% CI 15.8–36.3) exceeded 10 s. TUG correlated strongly and inversely with 6MWD (r = −0.75, 95% CI −0.84 to −0.61; *p* < 0.001). This remained unchanged after adjustment for age, sex, and BMI (r = −0.76) and held within men (r = −0.79) and women (r = −0.78) separately. Handgrip strength correlated with 6MWD (right r = 0.40; left r = 0.39; both *p* ≤ 0.001) and attenuated to non-significance after adjustment for sex (r = 0.22, *p* = 0.076) and for age, sex, and BMI (r = 0.18, *p* = 0.16). In multivariable regression, TUG was independently associated with 6MWD (−21.4 m per second, *p* < 0.001), whereas handgrip strength was not (*p* = 0.064). **Conclusions:** A substantial proportion of adults with T2DM in this cohort displayed low-physical-performance profiles. Simple performance batteries combining TUG, handgrip strength, and 6MWD warrant further study as a potential means of identifying patients with T2DM who could benefit from targeted rehabilitation.

## 1. Introduction

Type 2 diabetes mellitus (T2DM) is among the most prevalent chronic metabolic conditions worldwide and is increasingly associated with accelerated physical decline that resembles physical frailty seen in older populations and other chronic diseases. Physical frailty is classically defined by a phenotype of muscle weakness, slow gait, low physical activity, exhaustion, and unintentional weight loss [1]. Patients with T2DM are particularly susceptible to the muscular component of this phenotype: chronic hyperglycaemia, insulin resistance, microvascular disease, and low-grade systemic inflammation adversely affect skeletal muscle mass, strength, and function, even in the absence of overt diabetic complications [2,3]. This relationship appears bidirectional, with reduced muscle mass and quality, sometimes termed diabetic sarcopenia, also contributing to worsening glycaemic control and further functional decline [4,5].

Several convergent mechanisms have been proposed to explain this pattern of accelerated functional decline, including the accumulation of advanced glycation end-products in skeletal muscle, impaired anabolic signalling, microvascular rarefaction, and chronic low-grade inflammation [3,4,5]. Physical frailty and diabetes frequently coexist in older adults and share overlapping pathophysiological pathways, prompting calls for more routine frailty screening within diabetes care pathways [6,7,8]. However, physical frailty remains an important but under-recognised contributor to disability, hospitalisation, and mortality in chronic disease populations and is rarely assessed in routine diabetes care. Simple physical performance tests offer a pragmatic alternative [9,10]. The Timed Up and Go (TUG) test is a brief, standardised measure of functional mobility that integrates lower-limb strength, gait speed, balance, and cognitive processing [11]. Handgrip strength (HGS) is a well-established, low-cost surrogate for whole-body muscle strength and is best measured using a standardised protocol to ensure reliability across settings [12], with the important qualification that its interpretation is strongly sex-dependent [13]. The six-minute walk distance (6MWD) provides a complementary, objective index of submaximal functional exercise capacity, assessed according to standardised guidelines [14].

While TUG-based physical frailty screening has been validated in COPD and older community-dwelling adults [9,10], comparatively little is known about how TUG, handgrip strength, and 6MWD relate to one another and how many individuals with T2DM meet performance-based mobility thresholds for physical frailty risk. This study aimed to characterise physical performance using TUG, HGS, and 6MWD in a cohort of adults with T2DM to quantify the proportion of participants exceeding exploratory TUG-based mobility thresholds at both an 8 s and a 10 s cutoff and to examine the relationships among these measures and basic demographic and anthropometric characteristics.

## 2. Materials and Methods

### 2.1. Study Design and Participants

This was a cross-sectional study of adults with a physician-confirmed diagnosis of T2DM, diagnosed in accordance with American Diabetes Association criteria (fasting plasma glucose ≥ 126 mg/dL, random plasma glucose ≥ 200 mg/dL, or glycated haemoglobin ≥ 6.5%), with a disease duration exceeding five years. Participants with thoracic or musculoskeletal deformities, unstable cardiovascular disease, severe neurological conditions, or other conditions likely to independently impair mobility or grip strength were excluded, consistent with the exclusion criteria applied in related work by our group in cardiometabolic populations. Sixty-five adults met these criteria and form the analytic sample. These exclusions were deliberately broad and are likely to have yielded a cohort healthier than the general T2DM population, so any impairment detected here probably understates that present in routine practice. The study is reported with reference to the STROBE statement [15].

### 2.2. Timed Up and Go Test

The TUG was performed using a standard chair with arm rests. Participants were seated with their backs against the chair and, on the command “go,” stood up, walked three metres at a comfortable pace, turned, walked back, and sat down again; the time to complete the task was recorded in seconds using a stopwatch. Participants were categorised as exceeding or not exceeding TUG values of 8 and 10 s. These values are used as exploratory external reference thresholds and not as diagnostic criteria: neither has been validated against a criterion frailty standard in adults with T2DM, and both were derived predominantly in older or COPD populations [9,10].

### 2.3. Handgrip Strength

Maximal handgrip strength was measured for the right and left hand using a hand dynamometer, with participants seated and the elbow flexed at 90 degrees; the higher of two attempts per hand was retained for analysis [12]. Because grip strength is strongly sex-dependent [13], values are additionally reported separately by sex, together with the proportion below the sex-specific consensus thresholds for low grip strength defined by EWGSOP2 (<27 kg men, <16 kg women) [16] and AWGS 2019 (<28 kg men, <18 kg women) [17].

### 2.4. Six-Minute Walk Distance

Functional exercise capacity was assessed using the 6MWD test performed over a flat, standardised indoor course, with standardised encouragement provided throughout, in line with American Thoracic Society methodology [14]. The total distance walked in six minutes was recorded in metres. A distance below 350 m was applied as an exploratory external threshold rather than a diagnostic criterion; this value derives from the SOLVD trial of patients with left ventricular dysfunction [18] and has not been validated in T2DM.

### 2.5. Statistical Analysis

Data analysis was performed using IBM SPSS Statistics for Windows, Version 27.0 (IBM Corp., Armonk, NY, USA). Continuous variables were summarised as mean ± standard deviation. Proportions exceeding each TUG threshold are reported with Wilson 95% confidence intervals. Between-group comparisons used Welch’s *t*-test, with mean differences, 95% confidence intervals, and Cohen’s d reported alongside *p*-values. Pearson correlation coefficients are reported with 95% confidence intervals derived by Fisher z-transformation. Because handgrip strength and 6MWD are strongly sex- and body-size-dependent [13], partial correlations adjusted for sex and for age, sex, and BMI are reported alongside the unadjusted coefficients, together with sex-stratified correlations and a multiple linear regression modelling 6MWD from TUG, handgrip strength, age, sex, and BMI simultaneously. Agreement between the TUG- and 6MWD-based threshold categorisations was assessed by cross-tabulation and Cohen’s κ with a bootstrap 95% confidence interval. Statistical significance was set at *p* < 0.05.

## 3. Results

### 3.1. Participant Characteristics

Sixty-five adults with T2DM were included (Table 1). The sample had a mean age of 55.6 ± 4.8 years and was predominantly male (64.6%), with a mean BMI of 29.6 ± 4.4 kg/m^2^ in the overweight-to-obese range. Mean right and left handgrip strength was 30.8 ± 10.1 kg and 28.0 ± 9.3 kg, respectively. Mean TUG time was 9.30 ± 3.02 s, and mean 6MWD was 401.2 ± 96.3 m.

### 3.2. Proportions Exceeding Published Mobility Thresholds

Forty-four participants (67.7%; 95% CI 55.6–77.8) recorded a TUG time of 8 s or more, and 21 (32.3%) did not. At the more conservative 10 s threshold, 16 participants (24.6%; 95% CI 15.8–36.3) exceeded the threshold and 49 (75.4%) did not. These proportions describe performance relative to externally derived thresholds; they are not estimates of frailty prevalence, and participants are not described as frail or non-frail.

Participants exceeding the 8 s threshold were significantly older than those below it (mean difference 2.8 years, 95% CI 0.2 to 5.4; *p* = 0.039, d = 0.59) and walked substantially less far in six minutes (difference −79.7 m, 95% CI −122.8 to −36.6; *p* = 0.001, d = −0.89). At the 10 s threshold, the age difference did not reach significance (*p* = 0.073), whereas the 6MWD difference was large (difference −124.3 m, 95% CI −179.4 to −69.2; *p* < 0.001, d = −1.55). Body mass index and handgrip strength did not differ significantly between subgroups at either threshold (Table 2).

### 3.3. Agreement Between the Two Threshold-Based Categorisations

Nineteen participants (29.2%) walked less than 350 m, and 16 (24.6%) exceeded the 10 s TUG threshold. Agreement was assessed by cross-tabulation and Cohen’s κ rather than by comparing percentages. Ten participants were common to both groups; observed agreement was 76.9%, and Cohen’s κ was 0.42 (bootstrap 95% CI 0.15–0.65), indicating fair-to-moderate agreement (Table 3).

### 3.4. Sensitivity of Proportions to Threshold Choice

**Figure 1:**
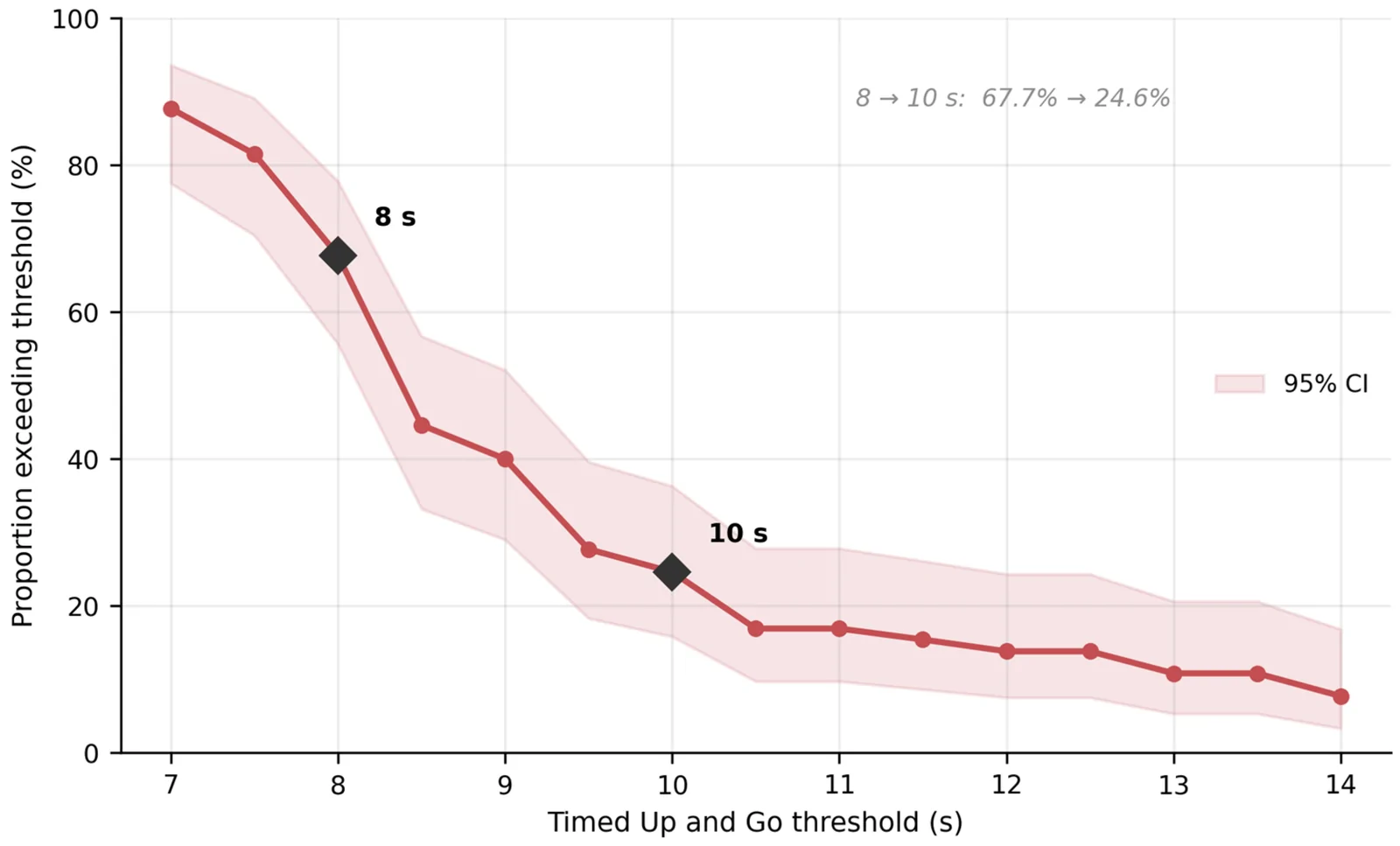
Proportion of participants exceeding Timed Up and Go thresholds between 7 and 14 s, plotted at 0.5 s increments with Wilson 95% confidence intervals (shaded). Diamonds mark the two thresholds most frequently cited in the literature.

Forty-four participants (67.7%; 95% CI 55.6 to 77.8) exceeded a TUG of 8 s, and 16 (24.6%; 95% CI 15.8 to 36.3) exceeded 10 s (Figure 1). Across the range examined, the proportion classified fell from 87.7% at 7 s to 81.5% at 7.5 s, 67.7% at 8 s, 44.6% at 8.5 s, 40.0% at 9 s, and 24.6% at 10 s. Participants exceeding either threshold walked substantially less far than those below it (8 s: difference −79.7 m, 95% CI −122.8 to −36.6, *p* = 0.001, d = −0.89; 10 s: difference −124.3 m, 95% CI −179.4 to −69.2, *p* < 0.001, d = −1.55), whereas handgrip strength and body mass index did not differ at either threshold. Nineteen participants (29.2%) walked less than 350 metres; ten of these also exceeded the 10 s TUG threshold, giving an observed agreement of 76.9% and Cohen’s κ of 0.42 (95% CI 0.15 to 0.65).

### 3.5. Sex-Stratified Performance

Men and women did not differ significantly in age, BMI, or TUG, but men had significantly higher handgrip strength and greater 6MWD (Table 4). Eight men (19%) and two women (9%) fell below the EWGSOP2 threshold for low grip strength; nine men (21%) and five women (22%) fell below the AWGS 2019 threshold.

### 3.6. Associations Between Physical Performance Measures

TUG was strongly and inversely correlated with 6MWD (r = −0.75, 95% CI −0.84 to −0.61, *p* < 0.001), and this association was essentially unchanged after adjustment for sex alone (r = −0.78), for age, sex, and BMI together (r = −0.76), and after additionally adjusting for handgrip strength (r = −0.77). Analysed separately within each sex, the association remained strong in both men (r = −0.79) and women (r = −0.78; Table 4). Handgrip strength was more modestly correlated with 6MWD for both the right hand (r = 0.40, 95% CI 0.17–0.58, *p* = 0.001) and the left hand (r = 0.39, *p* = 0.001). These associations attenuated after adjustment for sex (r = 0.22, *p* = 0.076; r = 0.21, *p* = 0.095) and further after adjustment for age, sex, and BMI (r = 0.18, *p* = 0.16 for both) (Table 5 and Table 6). Even within each sex, they were small and non-significant. TUG was not significantly associated with handgrip strength (right r = −0.13, *p* = 0.307; left r = −0.16, *p* = 0.198), BMI (r = 0.20, *p* = 0.118), or age (r = 0.19, *p* = 0.138).

## 4. Discussion

This study examined the interrelationships among three inexpensive performance tests in adults with T2DM and evaluated the behaviour of externally derived mobility thresholds in this population. Three observations warrant discussion: the TUG test was the measure most consistently associated with walking capacity; the association between handgrip strength and walking capacity was not independent of sex and BMI; and the proportion of participants categorised as impaired depended substantially on the threshold selected.

The coherence between the TUG test and the 6MWD is consistent with the small body of work that has measured both in diabetes. In a study of 156 participants comprising individuals with T2DM with and without polyneuropathy and healthy controls, lower-limb muscle strength was reduced in diabetes and correlated with performance on both the TUG test and the 6MWD test, with coefficients between 0.22 and 0.59 [19]. A more recent Saudi study of 95 adults with T2DM found that the one-minute sit-to-stand test predicted 6MWD distance after adjustment for age, sex, body mass index, physical activity, and diabetes duration [20]. Both reports point to the same conclusion reached here: tasks that require repeated weight-bearing loading of the lower limb carry information about walking capacity that survives statistical adjustment. Criterion evidence supports this interpretation, since 6MWD in T2DM correlates with directly measured maximal oxygen uptake [21], indicating that these field tests index a genuine capacity for sustained ambulatory work rather than a narrow motor skill. The two tests are not, however, interchangeable in the information they add: because they share a substantial proportion of their variance, administering both yields less incremental information than their separate labels suggest, and the shorter test imposes far lower spatial and temporal demands.

The dissociation of handgrip strength from both walking measures has a plausible physiological basis specific to diabetes. Diabetic polyneuropathy is length-dependent, affecting the longest axons first and therefore involving the distal lower limb before the upper limb. The investigators of the Health, Aging and Body Composition study advanced precisely this argument, noting that if neuropathy contributes to muscle dysfunction in diabetes, then the lower extremities should be affected preferentially [5]. Empirical data support this asymmetry: knee extensor and ankle plantar- and dorsiflexor strength are reduced in T2DM, with and without clinically evident polyneuropathy, and the severity of neuropathy correlates with TUG performance [22]. Proprioceptive loss and impaired postural control compound this effect through mechanisms largely independent of muscle force [19], and altered gait characteristics have been documented in diabetes irrespective of strength [22]. A grip dynamometer, by contrast, samples a single isometric contraction of the forearm and hand, imposing no demand on balance, gait control, or cardiorespiratory reserve and lying outside the territory in which length-dependent neuropathy first manifests [23,24]. It is therefore unsurprising that grip strength should track walking capacity weakly in a population whose functional limitation arises predominantly from the lower limb.

A second explanation operates alongside the first. Handgrip strength is among the most strongly sexually dimorphic of all physical measures, and in the present cohort, men and women differed markedly in grip strength while not differing at all in mobility. Under these conditions an unadjusted correlation between grip strength and any sexually dimorphic outcome will partly express the separation between male and female distributions rather than a relationship operating within individuals, which is why the coefficient contracted once sex was modelled and did not reach significance within either sex. This is a general measurement problem rather than a feature of this dataset, and it has been identified as a limitation of interpreting grip strength without sex-specific reference values [13]. The observation does not diminish the standing of grip strength as a clinical measure. Its prognostic value for mortality is well established, each 5 kg decrement being associated with higher all-cause and cardiovascular death in an international cohort of 139,691 adults [24], and reduced grip strength in T2DM relative to controls has been demonstrated with adjustment for age, smoking, obesity, physical activity, and protein intake [23]. Grip strength also remains a required component of sarcopenia case-finding [3,4,25,26]. The distinction is between a measure whose prognostic value for long-term risk is well established in the wider literature [24] and a measure that explains present ambulatory function [27,28]. Our cross-sectional data speak only to the latter and cannot address long-term outcomes, which were not examined; we therefore make no claim that grip strength is superior for predicting long-term risk.

These findings bear on the construct this study set out to examine. Slowness and weakness are both components of the physical frailty phenotype [2], and it is often assumed that measures of each are broadly substitutable indicators of the same underlying decline. In this cohort they were not. Mobility and upper-limb strength varied independently, which suggests that within the physical frailty phenotype, the slowness and weakness domains may diverge in T2DM and that a measure of one cannot stand in for the other. This has a direct implication for how the phenotype should be operationalised in diabetes: a screening approach that relies on grip strength alone risks failing to identify individuals whose limitation is locomotor in origin. It should be emphasised that this reasoning concerns the performance-based components of physical frailty only. Exhaustion, habitual physical activity, and unintentional weight loss were not recorded, multisystem frailty defined by deficit accumulation [4] was not assessed, and no participant in this study can be described as frail. What the data support is a statement about the internal structure of physical performance, not about frailty status.

The instability of threshold-based classification reinforces this restraint. Both of the values applied here originate in populations older than the present cohort [9,10], and normative data indicate that mean performance among healthy adults aged 60 to 69 years already approaches the lower of them [29]. A proportion derived from such a threshold therefore describes the position of the cohort relative to an external reference rather than the prevalence of any condition, and the magnitude of the change across a narrow range of plausible values makes any single figure difficult to defend. Similar caution applies to the 6MWD. Distance alone does not capture the physiological cost of walking, and preserved distance may coexist with reduced reserve [21].

The contribution of this analysis lies in the direct comparison it permits. Previous work in T2DM has established that physical performance is reduced relative to controls, that it declines over time, and that it can feasibly be measured in routine care. What has not been examined is which of the commonly co-administered tests retains explanatory value when they are analysed together and adjusted for the demographic and anthropometric variables that influence all of them. Studies that have measured mobility and strength concurrently in this population have generally quantified lower-limb strength using dynamometry [21,27,28], which is informative mechanistically but impractical in most clinics, whereas the tests compared here require only a chair, a stopwatch, a corridor, and a handheld dynamometer. Demonstrating that the mobility measure retains its association after adjustment while the grip measure does not and that this holds within each sex provides an empirical basis for prioritising one test over another under realistic resource constraints. The finding is specific to explaining walking capacity and should not be generalised to other outcomes [30].

Several limitations qualify these conclusions. The cross-sectional design precludes inference about temporality or causation, and the mutual dependence of mobility and walking capacity cannot be resolved into a directional relationship. Physical frailty was not assessed with a validated instrument, and muscle mass was not measured, so the study characterises the performance domain alone. The clinical variables most likely to influence the associations reported, including diabetes duration, glycated haemoglobin, complications, comorbidity, treatment, and habitual physical activity, were unavailable and could not be modelled; residual confounding cannot be excluded, and the contribution of polyneuropathy to the dissociation described above could not be tested directly because neuropathy status was not recorded. No age- and sex-matched comparator group without diabetes was recruited, so the extent to which the observed profile is specific to T2DM cannot be determined, and benchmarking against published normative data is subject to differences in population, protocol, and era. Finally, the single-centre design and broad exclusion criteria are likely to have produced a cohort with better function than the wider T2DM population, which would attenuate rather than exaggerate the associations reported.

## 5. Conclusions

In adults with T2DM, the TUG test was the measure most consistently associated with functional walking capacity, and this association was independent of age, sex, and BMI and consistent across sexes. Handgrip strength, although a valuable prognostic marker and a required element of sarcopenia assessment, showed a weaker association that was largely attributable to shared dependence on sex and BMI and was attenuated to non-significance after adjustment for sex and BMI and did not reach significance within either sex. The divergence between these measures indicates that the slowness and weakness components of the physical frailty phenotype are not interchangeable in this population and is consistent with the length-dependent, lower-limb predominance of diabetic neuromuscular involvement.

Where a single brief performance test can be accommodated in routine diabetes review, these data support the selection of a lower-limb weight-bearing task such as the TUG test, which requires minimal space and equipment and retains its association with walking capacity after adjustment. Proportions generated by applying published mobility thresholds should be reported across a range of values rather than at a single cut-point and interpreted as describing performance relative to an external reference rather than as estimates of frailty prevalence. Walking distance requires population-appropriate reference equations if percent-predicted values are to be meaningful.

Because multidimensional frailty and muscle mass were not measured and the design was cross-sectional, these findings characterise physical performance and are hypothesis-generating with respect to frailty. Prospective studies incorporating a validated physical frailty phenotype assessment, objective body composition measurement, documentation of neuropathy and glycaemic status, an age- and sex-matched comparator group, and follow-up for falls and disability are required to establish whether performance-based screening identifies adults with T2DM who would benefit from early rehabilitative intervention and to derive thresholds appropriate to this population rather than borrowed from older cohorts.

## Figures and Tables

**Table 1:** Participant demographic, anthropometric, and physical performance characteristics.

Variable	Mean ± SD (or n, %)
N	65
Age (years)	55.6 ± 4.8
Sex, male:female	42:23 (64.6% male)
BMI (kg/m^2^)	29.6 ± 4.4
Weight (kg)	81.9 ± 14.8
Handgrip strength, right (kg)	30.8 ± 10.1
Handgrip strength, left (kg)	28.0 ± 9.3
Timed Up and Go (s)	9.30 ± 3.02
Six-minute walk distance (m)	401.2 ± 96.3

**Table 2:** Comparisons by TUG threshold, with mean differences, 95% confidence intervals, and Cohen’s d.

Variable	n (≥:<)	At/Above	Below	Mean Diff. (95% CI)	*p*; d
Age, ≥8 s	44:21	56.5 ± 4.5	53.7 ± 5.0	+2.8 (+0.2 to +5.4)	0.039; +0.59
BMI, ≥8 s	44:21	30.0 ± 4.4	28.9 ± 4.2	+1.1 (−1.2 to +3.4)	0.329; +0.26
HGS right, ≥8 s	44:21	29.8 ± 9.3	32.7 ± 11.8	−2.9 (−8.8 to +3.1)	0.335; −0.28
HGS left, ≥8 s	44:21	26.5 ± 8.1	31.0 ± 11.2	−4.4 (−10.0 to +1.2)	0.117; −0.48
6MWD, ≥8 s	44:21	375.4 ± 96.3	455.1 ± 72.4	−79.7 (−122.8 to −36.6)	0.001; −0.89
Age, ≥10 s	16:49	57.1 ± 3.3	55.1 ± 5.2	+2.0 (−0.2 to +4.3)	0.073; +0.43
BMI, ≥10 s	16:49	29.9 ± 4.4	29.6 ± 4.4	+0.3 (−2.3 to +2.9)	0.813; +0.07
HGS right, ≥10 s	16:49	28.5 ± 9.1	31.5 ± 10.4	−3.0 (−8.6 to +2.5)	0.276; −0.30
HGS left, ≥10 s	16:49	26.4 ± 8.7	28.5 ± 9.6	−2.0 (−7.3 to +3.3)	0.439; −0.22
6MWD, ≥10 s	16:49	307.4 ± 97.0	431.8 ± 74.4	−124.3 (−179.4 to −69.2)	<0.001; −1.55

Welch’s *t*-test. Values are mean ± SD. HGS, handgrip strength; 6MWD, six-minute walk distance.

**Table 3:** Cross-tabulation of TUG ≥10 s and 6MWD < 350 m.

	6MWD < 350 m	6MWD ≥ 350 m	Total
TUG ≥10 s	10	6	16
TUG <10 s	9	40	49
Total	19	46	65
Observed agreement 76.9%; κ = 0.42 (95% CI 0.15 to 0.65)			

**Table 4:** Sex-stratified physical performance.

Variable	Men (n = 42)	Women (n = 23)	*p*
Age (years)	55.7 ± 5.1	55.4 ± 4.4	0.805
BMI (kg/m^2^)	29.0 ± 4.0	30.7 ± 4.8	0.156
TUG (s)	9.1 ± 2.7	9.6 ± 3.6	0.544
HGS right (kg)	35.2 ± 9.5	22.7 ± 5.0	<0.001
6MWD (m)	428.6 ± 89.9	351.0 ± 88.5	0.002
Below EWGSOP2 grip threshold, n (%)	8 (19%)	2 (9%)	—
Below AWGS grip threshold, n (%)	9 (21%)	5 (22%)	—
TUG − 6MWD, r (*p*)	−0.79 (<0.001)	−0.78 (<0.001)	—
HGS right − 6MWD, r (*p*)	0.22 (0.167)	0.28 (0.205)	—

**Table 5:** Unadjusted and adjusted associations of TUG and handgrip strength with 6MWD.

Model	TUG r (95% CI)	*p*	HGS Right r (95% CI)	*p*
Unadjusted	−0.75 (−0.84 to −0.61)	<0.001	0.40 (0.17 to 0.58)	0.001
Adjusted for sex	−0.78 (−0.86 to −0.65)	<0.001	0.22 (−0.03 to 0.44)	0.076
Adjusted for age, sex, BMI	−0.76 (−0.85 to −0.63)	<0.001	0.18 (−0.08 to 0.41)	0.162
Adjusted for the other test	−0.77 (−0.85 to −0.63)	<0.001	0.19 (−0.06 to 0.42)	0.130

**Table 6:** Multiple linear regression modelling six-minute walk distance (N = 65).

Predictor	β (Unstandardised)	95% CI	*p*
Timed Up and Go (s)	−21.39	−26.05 to −16.74	<0.001
Handgrip strength, right (kg)	+1.79	−0.11 to +3.68	0.064
Age (years)	+0.04	−3.20 to +3.28	0.979
Sex (male)	+37.43	−0.31 to +75.17	0.052
BMI (kg/m^2^)	−4.02	−7.25 to −0.79	0.016

β, unstandardised coefficient; Model R^2^ = 0.711, adjusted R^2^ = 0.686, F(5,59) = 28.96, *p* < 0.001, residual standard error 54.0 m. Diagnostics: Shapiro–Wilk W = 0.984 (*p* = 0.541); Breusch–Pagan *p* = 0.862; Durbin–Watson 1.77; maximum Cook’s distance 0.181; maximum studentised residual 2.37.

## Data Availability

The datasets used and/or analyzed during the current study are available from the corresponding author upon reasonable request.
